# Newer Oral Anticoagulant Agents: A New Era in Medicine

**DOI:** 10.2174/157340312801784934

**Published:** 2012-05

**Authors:** Ramil Goel, Komandoor Srivathsan

**Affiliations:** Division of Cardiovascular Diseases, Mayo Clinic, Phoenix, Arizona

**Keywords:** Acute coronary syndrome, apixaban, atrial fibrillation, dabigatran, deep venous thrombosis, pulmonary embolism, rivaroxaban, venous thromboembolism.

## Abstract

After a gap of almost 60 years following the development of warfarin, 2 new categories of oral anticoagulant agents have been approved for clinical use – the direct thrombin inhibitors and factor Xa inhibitors. These agents promise to be more convenient to administer with fixed dosing but still have equivalent efficacy and improved bleeding risk compared to warfarin. The clinical community is looking forward to the widespread usage of these agents but there is also some apprehension regarding bleeding risks, non-availability of specific reversal strategies and lack of specific monitoring parameters. This review article will attempt to educate the reader about three representative drugs from these classes: Dabigatran, Rivaroxaban and Apixaban. We will discuss the historical perspective to the development of these drugs, available research data and pharmacology of these agents. The best strategies for monitoring and reversal of these drugs in special situations will also be touched upon.

## INTRODUCTION AND HISTORICAL PERSPECTIVE

Therapeutic anticoagulation is widely used to treat and prevent thromboembolic disorders. Anticoagulant agents not only prevent new clot formation but also facilitate intrinsic mechanisms of clot lysis by retarding existing clot progression. 

Effective anticoagulation has formed the basis of treatment for acute venous thromboembolic (VTE) events (deep venous thrombosis and pulmonary embolism) for a long time and reduces the mortality rate in this condition from 30% to 3-8% [1, 2]. Anticoagulants are administered in a preventative role to reduce clot formation in inherited and acquired hypercoagulable states. Anticoagulation is also used to prevent clot formation related to atrial fibrillation (left atrial appendage) and those caused by foreign bodies in contact with blood stream (artificial valves, catheters and cardiac devices). Anticoagulation reduces the incidence of stroke in atrial fibrillation by 60% [3-5]. Anticoagulation for deep venous thrombosis (DVT) prophylaxis in hospitalized medical patients decreases the incidence of DVT by up to 67% [5].

The introduction of heparin in the 1930s was a major breakthrough and provided the first widely available anticoagulant agent [6]. The major limitation was its limited mode of administration as a parenteral only agent which required close monitoring. This was partially overcome by another drug of historical importance which was also was the first oral anticoagulant agent – warfarin. It was first synthesized in 1940 and named after the Wisconsin Alumni Research Foundation [7]. Warfarin and its congeners were the only available oral anticoagulant agents until recently. Even though it has immense efficacy as an anticoagulant, warfarin is universally acknowledged as a cumbersome agent to use. It has a delayed onset of action, unpredictable efficacy affected by genetics, co-administered drugs & diet, body weight and age of the patient. It requires periodic monitoring to ensure therapeutic levels and despite careful follow up, only about 50% of the patients are able to achieve therapeutic level as defined by international normalized ratio (INR) [8]. The fact that heparin and warfarin are in wide use, some 80 and 60 years after their respective discoveries, is a testament to the relative efficacy and safety of these drugs. On the other hand, it indicates a failure to develop more effective and improved anticoagulant agents. The ideal anticoagulant agent needs to be efficacious, safe, convenient to use and easy to administer (preferably be oral). 

Our improved knowledge of pharmacology and coagulation pathways has allowed us to develop newer anticoagulants which have shown significant promise. Beginning in the 1980s the low molecular weight heparins, enoxaparin and dalteparin being the principal agents, and then selective indirect factor Xa inhibitors like fondaparinux have been introduced [9]. These agents are parenteral and have their own limitations but nonetheless are seen as increasingly viable options to heparin. Danaparoid is another heparinoid with a mechanism of action similar to heparin, is an artificially formulated mixture of non-heparin glycosaminoglycans. This agent had been used extensively in patients with HIT after approval in 1996, but has not been available for use in the United States since 2002 after withdrawal by the manufacturer. Perhaps, their use primarily for HIT was being supplanted by emerging agents. Parenteral direct thrombin inhibitors like bivalirudin and argatroban were both approved in 2000 for unstable angina and heparin-induced thrombocytopenia respectively, are now being approved for expanding indications.

Newer oral anticoagulant agents have begun to emerge only recently and promise significant advantages over warfarin. These agents can be principally divided into two groups – direct thrombin inhibitors and oral factor Xa inhibitors. The first agent to be marketed in the first group was Ximelagatran and after 2 decades of development it was approved for use in DVT prophylaxis in 7 European countries by 2004. Despite showing significant efficacy, it never received FDA approval due to concerns about significant hepatotoxicity in a small proportion of patients. It was withdrawn from all markets in 2006 due to increasing reports of hepatotoxicity. The experience with this agent led to the development of another drug in the same class namely dabigatran. 

The other class of upcoming oral anticoagulants belongs to a different group of molecules that directly inhibit factor Xa. They are termed direct as they do not require antithrombin III to mediate their interaction with factor Xa. The clinically studied agents in this class include Rivaroxaban and Apixaban. The other agents in this class which have not yet completed phase 3 trials include edoxaban, otamixaban, betrixaban, darexaban, TAK 442 and YM 466.

**Fig. (1):**
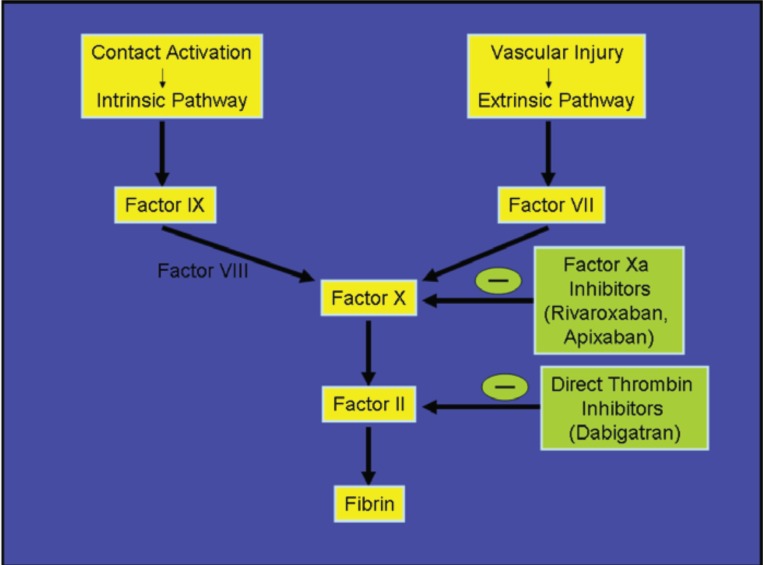
The coagulation pathway and points of action of the newer oral anticoagulant agents.

This review will discuss and compare the known pharmacology and available clinical data on the three oral agents which have been recently studied in widely publicized, large scale human trials and have generated great excitement. These agents include Dabigatran, Apixaban and Rivaroxaban. A single target in the coagulation cascade, fixed dosing independent of weight and generally of renal function, independence from monitoring coagulation effects and rapid onset of action mark the common benefits of these drugs. However, there are significant differences in their pharmacologic profile and approved indications. This focused review hopes to familiarize the reader with some of these issues pertaining to each drug. Their role in the interruption of the coagulation cascade is schematically displayed in Fig. **1**.

## DABIGATRAN

Dabigatran was first synthesized in the lab by Norbert H. Hauel *et al* for Boerhinger Ingelheim in 2002. It is a potent, reversible inhibitor of both free and clot-bound thrombin. The 1st clinical study in 2004 showed non-inferiority to enoxaparin in DVT prevention [10]. It is administered as a prodrug called dabigatran etexilate which is converted to the active compound dabigatran by serum esterases. Its bioavailability is of the order of 6-7% and the time to onset of action is about 30 minutes. 1/3^rd^ of the drug is protein bound and thus this part is non-dialyzable. The excretion is primarily renal (80%) and half life varies from 9-16 hours depending upon age of the subject. Plasma concentration of dabigatran does not depend on liver metabolism but it is increased or decreased by inhibitors or inducers of the p-glycoprotein transporter respectively. Amiodarone, dronedarone, quinidine, ketoconazole and verapamil being p-glycoprotein inhibitors raise levels of dabigatran. Rifampin being an inducer can decrease dabigatran levels. See Table **1** for pharmacologic summary and comparison with the other new anticoagulant agents.

### Available Evidence

#### 1 VTE prophylaxis:

The RE-NOVATE (Dabigatran etexilate versus enoxaparin for prevention of venous thromboembolism after total hip replacement) and RE-MODEL (Oral dabigatran etexilate vs. subcutaneous enoxaparin for the prevention of venous thromboembolism after total knee replacement) studies established the non-inferiority of dabigatran compared with enoxaparin at a dose of 40 mg sq once daily or 30 mg sq twice daily for VTE prophylaxis after hip and knee replacement surgeries respectively [11, 12]. The doses used were 75-110 mg of dabigatran started 1-4 hours post-operatively followed by 75-220 mg daily. Patients with total knee replacement (TKR) were treated for 6-10 days and those with total hip replacement (THR) were treated for 28-35 days. 

The later administration of dabigatran (6-12 hours post-operatively) at a dose of 75-220 mg once daily has, however, been shown to be inferior to enoxaparin at a dose of 30 mg SQ twice daily (given after similar period post-operatively) as shown in the RE-MOBOLIZE (The oral thrombin inhibitor dabigatran etexilate vs the North American enoxaparin regimen for the prevention of venous thromboembolism after knee arthroplasty surgery) trial [13]. The VTE incidence was 31% in the enoxaparin group vs. 34% in the dabigatran group [13]. A pooled analysis of all three trials, however, did not show any difference in the primary outcomes of VTE related death or VTE events between the dabigatran and enoxaparin groups [14]. There was no difference in major bleeding complications in any of the trials.

#### 2 VTE treatment:

The RE-COVER (Dabigatran versus Warfarin in the Treatment of Acute Venous Thromboembolism) trial proved non-inferiority of dabigatran 150 mg twice daily compared to warfarin for long term anticoagulation after patient with an acute VTE event had received 5-10 days of parenteral anticoagulation with IV heparin or SQ enoxaparin [15]. No difference in major bleeding rate was observed. Dabigatran has not been approved by the FDA for this indication yet. Dabigatran has a very rapid onset of action and potentially can be used for the acute treatment of VTE without need for initial parenteral heparin administration. However, it has not been studied in that role. 

#### 3 Atrial Fibrillation:

The RE-LY (Randomized Evaluation of Long-Term Anticoagulation Therapy) trial compared dabigatran at a dose of 110-150 mg twice daily with dose adjusted warfarin in 18,113 patients with non-valvular atrial fibrillation (average CHADS_2_ score = 2.1) [16]. At a dose of 110 mg of dabigatran there was no difference in cerebrovascular accidents (CVA) between the 2 groups but a significant advantage of dabigatran over warfarin was seen in terms of major bleeding and intracranial hemorrhage. At a dose of 150 mg, dabigatran was superior to warfarin in preventing CVAs but its advantage in terms of preventing major bleeding events was lost even though the specific benefit in preventing intracranial hemorrhage was maintained. See Table **2**.

#### 4 Mechanical Valves:

No trial to date has been published assessing the use of dabigatran for use in this patient population. Animal studies show promise [17, 18].

#### 5 Acute Coronary Syndrome:

Use of dabigatran in the setting of acute coronary syndrome as the anticoagulant of choice has not been evaluated. Its use to assess safety in those with a recent myocardial infarction (MI) to prevent recurrence of MI over a 6 month period was studied in the RE-DEEM (Dabigatran vs. placebo in patients with acute coronary syndromes on dual antiplatelet therapy: a randomized, double-blind, phase II trial) trial [19]. In this setting its use was associated with a dose-dependent increase in bleeding events of up to 2-4 times that seen in the placebo group. As this trial was not designed to look at outcomes in terms of cardiovascular effects, the net clinical benefit is unknown.

### Drug Dosing

The FDA approved adult dabigatran dose for non-valvular atrial fibrillation is 150 mg twice daily. The dose for renally impaired (creatinine clearance, CrCl 15-30 ml/min) patients is 75 mg twice daily based on pharmacokinetic modeling. The dose for CrCl <15 ml/min is not defined.

The patients receiving dabigatran for post-operative prophylaxis should be administered 220 mg once daily, but dose should be restricted to 110 mg when prescribing it between 1-4 hours post-operatively. The duration of therapy for knee replacement patients is 10 days. That for hip replacement patients is 28-35 days.

### Adverse Effects and Issues with Usage

Apart from bleeding complications including intracranial hemorrhage, gastrointestinal hemorrhage, hematuria, dyspepsia is the major side effect of dabigatran. 

The relationship of dabigatran levels with aPTT (activated partial thromboplastin time) is non-linear. However at lower doses use of dabigatran does correlate with a higher aPTT and can be used to assess compliance.

Thrombin time gives a fair qualitative idea of the level of anticoagulation with dabigatran. Ecarin clotting time has a linear relation with dabigatran levels but is not recommended for routine clinical use.

No good antidote exists to reverse dabigatran effects in life-threatening bleeding situations like intracranial hemorrhage. Waiting for about 4 half lives (2 days) would result in elimination of most of the drug and its effect. For faster extraction of drug, hemodialysis is a viable option and can get rid of 60% of the drug in 2-3 hours. Prothrombin complex concentrates (PCC) which contain all or most of the vitamin K-dependent clotting factors have been shown to rapidly reverse dabigatran’s anticoagulant effects [20]. Their use is associated with a finite risk of precipitating thromboembolism and should be avoided in those with aPTT patient:control ratio of ≤ 1. Recombinant factor VII is another potential option for reversal but so far has shown inconsistent performance in this setting.

## RIVAROXABAN

Rivaroxaban is a small molecule direct inhibitor of factor Xa and is administered in its active form. It has excellent bioavailability at 80% when taken orally. It has a rapid onset of action with maximal inhibition of factor Xa noted within 1-4 hours of ingestion. About 95% of the drug is protein bound making dialysis useless to achieve rapid elimination of drug. The half life of the drug is 7-13 hours (with higher duration with increasing age). A third of the drug is excreted unchanged in the urine (as active form); the remaining 2/3^rd^ is metabolized in the liver and half of this part is excreted in the urine and half in the feces. The hepatic metabolism of the drug does depend on the CYP3A4 system and makes it prone to interactions with drugs that use this pathway. Drugs such as ketoconazole (and congeners), ritonavir which inhibit CYP3A4 can cause rise in rivaroxaban levels. Similarly, inducers of this enzyme like Rifampin, carbamazepine and phenytoin can lead to a drop in rivaroxaban levels. Like dabigatran, rivaroxaban elimination also depends on the p-glycoprotein system and interactions with drugs inducing or inhibiting p-glycoprotein system are possible. See Table **1** for pharmacologic summary and comparison with the other new anticoagulant agents.

### Available Evidence

#### 1 VTE prophylaxis:

The phase III RECORD ((REgulation of Coagulation in ORthopedic Surgery to Prevent Deep Venous Thrombosis and Pulmonary Embolism) studies comprised of 4 separate trials. The RECORD1 trial compared 10 mg of rivaroxaban daily (starting 6-8 hours post-operatively after THR) to 40 mg SQ of enoxaparin administered for 31-39 days [21]. The RECORD 3 study was similar except done in post-operative TKR patients with anticoagulation administered for 10-14 days [22]. In both studies rivaroxaban was superior to enoxaparin in terms of preventing a VTE event. The RECORD 4 trial compared 10 mg daily of rivaroxaban with 30 mg sq twice daily of enoxaparin in post-operative patients following TKR [23]. Rivaroxaban was superior to enoxaparin even at this dose in terms of achieving the primary end point of proximal DVT, non-fatal PE and all-cause mortality. The MAGELLAN (Multicenter, randomized, parallel Group Efficacy and safety study for the prevention of VTE in hospitalized medically iLL patients comparing rivaroxabAN with enoxaparin) trial, not published yet, compared enoxaparin 40 mg sq daily for 10 days with rivaroxaban 10 mg once daily for 35 days in the setting of acute medical illness. This study did not find any difference between enoxaparin and rivaroxaban in terms of the primary end-point (a composite of asymptomatic proximal DVT detected by ultrasonography, symptomatic DVT, symptomatic nonfatal pulmonary emboli, and VTE-related death). However, the risk of clinically relevant bleeding was doubled in the rivaroxaban group. 

#### 2 VTE treatment:

The EINSTEIN-DVT (Oral rivaroxaban versus standard therapy in the initial treatment of symptomatic deep vein thrombosis and long-term prevention of recurrent venous thromboembolism) trial compared rivaroxaban with standard therapy in patients with acute DVT but without symptomatic pulmonary embolism (PE) [24]. Oral rivaroxaban was given at a dose of 15 mg twice daily for the first 3 weeks, followed by 20 mg once daily for the intended 3 months of treatment. Standard therapy consisted of subcutaneous enoxaparin, 1.0 mg per kilogram of body weight twice daily (continued till 2 consecutive daily INR values of 2.0 were achieved) and either warfarin or acenocoumarol, started within 48 hours after randomization. The primary endpoint was symptomatic, recurrent VTE. Rivaroxaban had noninferior efficacy with respect to the primary outcome. No differences in safety outcomes were noted. 

#### 3 Atrial Fibrillation:

The ROCKET-AF (Rivaroxaban Once Daily Oral Direct Factor Xa Inhibition Compared With Vitamin K Antagonism for Prevention of Stroke and Embolism Trial in Atrial Fibrillation) trial was a double blind randomized control trial to assess the efficacy of rivaroxaban 20 mg daily in comparison to dose adjusted warfarin in patients with non-valvular atrial fibrillation [25]. The mean CHADS_2_ score of the patients was 3.5. Rivaroxaban was shown to be non-inferior to dose adjusted warfarin in terms of achieving the primary end-point. No differences in overall bleeding complications were noted between the 2 groups although significant reductions in fatal and intracranial bleeding were noted in rivaroxaban arm. See Table **2**.

#### 4 Mechanical Valves:

No human studies have been reported assessing the efficacy of rivaroxaban to prevent mechanical valve thrombosis, preliminary in-vitro studies have shown some promise [26].

#### 5 Acute Coronary Syndrome:

Rivaroxaban has been studied to prevent cardiovascular events in a secondary prevention role after a recent acute coronary event. The recently published ATLAS ACS 2–TIMI 51 (Rivaroxaban in Combination With Aspirin Alone or With Aspirin and a Thienopyridine in Patients With Acute Coronary Syndromes–Thrombolysis In Myocardial Infarction) trial recruited patients admitted for an acute coronary syndrome within the last 7 days [27]. It divided these patients into 3 equal groups: receiving 2.5 mg twice daily of rivaroxaban, 5 mg twice daily of rivaroxaban and placebo for a mean of 13 months. The 2.5 mg dose of rivaroxaban was associated with reduced cardiovascular (2.7% vs. 4.1%, P=0.002) as well as total mortality (2.9% vs. 4.5%, P=0.002). Rivaroxaban, however, did increase the rate of major bleeding episodes not related to coronary-artery bypass grafting (2.1% vs. 0.6%, P<0.001) and intracranial hemorrhage (0.6% vs. 0.2%, P=0.009).

### Drug Dosing

The FDA approved rivaroxaban dose for DVT prophylaxis is 10 mg once daily for a period of 12-14 days after TKR and 35 days after THR.

The FDA also approved rivaroxaban for stroke prophylaxis in atrial fibrillation on November 4, 2011 at dose of 20 mg once daily. The dose for patients with moderate renal impairment (CrCl 15-50 ml/sec) is 15 mg once daily. The drug is to be avoided for severe renal impairment (CrCl <15 ml/sec).

It is also recommended that the drug be avoided in moderate to severe hepatic impairment.

### Adverse Effects and Issues with Usage

Bleeding related to its anticoagulant effect is the most common adverse effect. Syncope and thrombocytopenia are uncommon effects. Routine monitoring of the anticoagulant effects of rivaroxaban is not recommended. However, the tests which correlate with its anticoagulation efficacy are prothrombin time (PT) and endogenous thrombin potential (ETP). 

Just like dabigatran, there is no specific antidote for rivaroxaban. Waiting for the effect of the drug to be eliminated from the system is the best option when feasible. Unlike dabigatran, rivaroxaban cannot be hemodialyzed as 95% of the drug in the serum is protein bound. However, using prothrombin complex concentrates is more effective in cases of acute life threatening bleeds related to rivaroxaban. A recent study showed complete reversal of anticoagulant effects of rivaroxaban with infusion of PCC [28]. In-vitro and animal studies have shown the potential benefit of recombinant factor VIIa in the reversal of rivaroxaban effects [29, 30].

## APIXABAN

Apixaban is another oral direct factor Xa inhibitor which has been studied in recent clinical trials. It is administered as an active compound and has rapid onset of action with a bioavailability of 50%. The peak plasma levels are achieved about 3 hours after oral administration. The drug is 87% protein bound. The half life is 8-15 hours and 75% elimination is via the liver after metabolism using the CYP 3A4 pathway. 25% of the metabolized drug is excreted renally. Inhibition of both the CYP 3A4 and p-glycoprotein can increase drug levels. See Table **1** for pharmacologic summary and comparison with the other new anticoagulant agents.

### Available Evidence

#### 1 VTE prophylaxis:

The ADVANCE-1 (Apixaban Dose Orally vs. Anticoagulation with Enoxaparin-1) trial compared apixaban 2.5 mg twice daily to enoxaparin 30 mg sq twice daily for VTE prophylaxis in patients undergoing TKR. These drugs were initiated 12-24 hours after surgery and were administered for 10-14 days [31]. There was no specific difference in the two groups in terms of the primary efficacy outcome of asymptomatic and symptomatic deep-vein thrombosis, nonfatal pulmonary embolism, and death from any cause during treatment. Apixaban group did have decreased incidence of clinically relevant bleeding. Apixaban, however, did not meet prespecified criteria for non-inferiority and the trial was inconclusive. The ADVANCE-2 and ADVANCE-3 trials compared apixaban 2.5 mg twice daily with enoxaparin 40 mg once daily in patients undergoing TKR and THR respectively. The enoxaparin was started 12 hours preoperatively and the apixaban started 12-24 hours post-operatively in both groups [32, 33]. The duration of treatment was 10-14 days in ADVANCE-2 and 35 days in ADVANCE-3. In both these trials apixaban was non-inferior to enoxaparin in terms of reaching the primary end-point, defined similarly as in ADVANCE-1.

#### 2 VTE treatment:

The role of apixaban in the treatment of an acute VTE event is being investigated under the ongoing AMPLIFY trial (http://clinicaltrials.gov/;NCT00643201).

#### 3 Atrial Fibrillation:

The AVERROS (Apixaban VERsus acetylsalicylic acid to pRevent strOkE in atrial fibrillation patientS who have failed or are unsuitable for vitamin K antagonist treatment) trial was a randomized control study which compared 5 mg twice daily of apixaban with 81-324 mg of aspirin in patients with atrial fibrillation not deemed to be candidates for Warfarin (mean CHADS_2_ score = 2) [34]. The primary outcome of stroke or systemic embolism was assessed over a mean follow up period of 1.1 years. The primary outcome was achieved in the aspirin group at a rate of 3.7% per year compared to 1.6% in the apixaban group (p<0.001). The rates of major bleed and intracranial hemorrhage were not statistically different. 

The ARISTOTLE (Apixaban for Reduction of Stroke and Other Thromboembolic Events in Atrial Fibrillation) trial compared apixaban with warfarin in patients with atrial fibrillation or flutter deemed to be high risk for stroke [35]. The average CHADS_2_ score of patients in this trial was 2.1. 18,210 patients received apixaban 5 mg twice daily or dose adjusted warfarin (for an INR =2-3) over an average follow up period of 1.8 years. This study showed a superiority of apixaban over warfarin in terms of reaching the primary end-point as defined by ischemic or hemorrhagic stroke or systemic embolism (1.27 % per year vs. 1.6 % per year, p <0.001 for non-inferiority and p <0.01 for superiority). The risk of bleeding or hemorrhagic stroke was also lower in the apixaban group (p<0.01, for both parameters). The all-cause mortality in the apixaban group was 3.52% vs. 3.94 % in the warfarin group (p=0.047). See Table **2**.

#### 4 Mechanical Valves: 

We are not aware of any published evidence of the use of apixaban in this setting at the time of writing of this manuscript.

#### 5 Acute Coronary Syndrome:

The APPRAISE (Apixaban for Prevention of Acute Ischemic and Safety Events) was a phase II study compared apixaban with placebo in patients with recent non-ST-elevation MI or with ST-elevation MI when administered for 6 months after the event in addition to current standard medical therapy [36]. This trial found a higher rate of bleeding in the apixaban arm, with a trend towards reduction in ischemic events over a follow up period of 6 months. After a reasonable dose had been determined from this trial, the APPRAISE II trial was undertaken comparing apixaban 5 mg twice daily to placebo in patients with recent high risk acute coronary syndrome [37]. This trial showed a higher risk of bleeding events with apixaban without any significant change in ischemic events.

### Drug Dosing

On November 29^th^ 2011, the FDA conferred on apixaban priority-review designation to expedite its review and potential approval. The drug doses are not yet formalized for this agent. The drug should be used with caution in patients with severe renal failure (CrCl 15-0 cc/min) and be avoided in those with end stage renal disease (CrCl < 15 cc/min).

### Adverse Effects and issues with usage

A package insert for apixaban is not yet available; most of the information regarding adverse effects can be estimated from the reported complications in the trials that have been conducted using apixaban. Bleeding seems to be the most common complication of apixaban use [35, 36]. The rest of the adverse effects were similar in the apixaban arm and the placebo arms in these studies. The issue of reversal of anticoagulant effects of the drug in emergency bleeds or prior to emergency surgeries has not been studied. Waiting for 3-4 half lives would be prudent if possible. Hemodialysis is not very effective due to the high degree of protein binding of the drug. Using prothrombin complex concentrates has been shown to be of use in preliminary studies[38]. Recombinant factor VIIa use maybe helpful but needs evaluation. 

## CLINICAL PERSPECTIVE AND PRACTICAL USE IN ATRIAL FIBRILLATION

Newer anticoagulants are non-inferior to warfarin in terms of systemic embolism and perhaps dabigatran 150 mg bid is marginally better. However, if a given patient’s INR is therapeutic most of the time, is it reasonable to switch to one of the newer agent? Taking all three trials in to consideration, the average time INR was therapeutic is in the 60% range. If the INRs have been therapeutic 70% of the time, will the non-inferiority margin be breached? Our own perspective is that if the INRs are therapeutic most of the time, then it is reasonable to continue warfarin. In patients with anticipated surgeries and bleeding issues, it is better to stay with warfarin. 

Which one of the newer agent is preferable? Based on available data, if the patients CHADS2 score is 3 or greater, it would be preferable to use rivaroxaban as it was studied in high risk population. Apixaban, on sub group analysis was less efficacious in younger patients (<65 Yrs) and Dabigatran related bleeding risk substantially increases with advancing age (>75 yrs). Potential for reversibility is better for factor Xa inhbitors (rivaroxaban and apixaban) than direct thrombin inhibitor (dabigatran).These factors may influence the choice of individual agent in a given patient.

Lastly, should rivaroxaban be given twice daily? Post market analysis may shed light on this issue.

## CONCLUSIONS

The newer oral anticoagulant agents have justifiably created significant excitement in the medical community. Major bleeding episodes appear to be less frequent in comparison to warfarin. Their use in stroke prophylaxis in atrial fibrillation for qualifying patients seems likely to become popular given the strong evidence. Their role in secondary prevention of MI after an acute coronary event is not justifiable at this point of time. The relative benefit of these agents in comparison to warfarin in terms of number needed to treat is displayed in Table **3**.

There do remain issues with their use. Monitoring for these agents is not perfected; even though they do not require routine monitoring; this may be of importance in critical situations like assessing bleeding risk prior to emergency surgery and during major life threatening bleeds. Reversal of these agents in these setting also has not been studied adequately and may make clinicians apprehensive in using them especially in patients with high bleeding risk. In summary, the introduction of these agents has added to the armamentarium of the physician important new classes of drugs. These pharmaceutical agents have potential benefits and significant risks. Their use requires familiarity with these drugs and good clinical judgment. 

## Figures and Tables

**Table 1.** Pharmacology of Newer Oral Anticoagulants in Comparison to Warfarin

	Dabigatran	Rivaroxaban	Apixaban	Warfarin
**Mechanism of Action**	Direct Thrombin inhibition	Direct Factor Xa Inhibition	Direct Factor Xa Inhibition	Reduced production of vitamin K dependent factors
**Bioavailability**	6-7%	80%	50%	>95%
**Half-Life**	9-16 h	7-13 h	8-15 h	40 h
**Dosing**	Fixed, once-twice daily	Fixed, once-twice daily	Fixed, twice daily	INR adjusted variable dosing
**Protein Bound fraction**	33%	95%	87%	99%
**Elimination**	80% renal	67% renal (half as inactive form)	25% renal (75% fecal)	Hepatic, primarily via CYP2C9
**Potential drug interactions**	Via p-glycoprotein modulating drugs	Via CYP 3A4 and p-glycoprotein modulating drugs	Via CYP 3A4 and p-glycoprotein modulating drugs	CYP2C9, CYP2C8, 2C18, 2C19, 1A2, and 3A4 modulating drugs
**Reversal Strategy**	Infusion of PCC (not as effective as in Rivaroxaban), administration of recombinant factor VIIa	Infusion of PCC, administration of recombinant factor VIIa	Infusion of PCC, administration of recombinant factor VIIa	Infusion of fresh frozen plasma, administration of vitamin K

**Table 2.** Trial Data on New Oral Anticoagulants for Stroke Prophylaxis in Atrial Fibrillation

Drug	Trial	Number of patients	Primary outcome	Primary outcome Drug vs. Warfarin (p-value)	Bleeding events Drug vs. Warfarin (p-value)	Remarks
Dabigatran 110 mg BID	RE-LY (14)	18,113	Stroke/ systemic embolism	1.53% vs. 1.69% (p<0.001 for noninferiority)	Major bleeding events only 2.71% vs. 3.36% (0.003)	Low dose of drug equal to warfarin in stroke prevention and superior in preventing major bleed
Dabigatran 150 mg BID				1.11% vs. 1.69% (p<0.001 for superiority)	Major bleeding events only 3.11 vs. 3.36% (0.31)	High dose better in preventing stroke, equal in causing major bleed
Rivaroxaban	ROCKET-AF (24)	14,264	Stroke/ systemic embolism	1.7% vs. 2.2% (<0.001 for noninferiority)	All reported bleeds 14.9 vs. 14.5 (0.44)	Rivaroxaban was superior to warfarin in preventing intra-cranial hemorrhage and fatal bleeding
Apixaban	ARISTOTLE (32)	18,201	Ischemic or hemorrhagic stroke/ systemic embolism	1.27% vs. 1.6% (p<0.001 for noninferiority, p = 0.01 for superiority)	Major bleeding events only 2.13% vs. 3.09% (<0.01)	Apixaban also had a mortality benefit over warfarin

**Table 3.** Relative Benefit Over Warfarin of the New Oral Anticoagulant Agents in Terms of Number Needed to Treat (NNT) to Prevent an Ischemic Stroke and Prevent a Major Bleed

Drug	Trial	NNT to prevent 1 ischemic stroke over warfarin per year	NNT to prevent 1 major bleeding episode over warfarin per year
Dabigatran 110 mg BID	RE-LY (14)	7	1.5
Dabigatran 150 mg BID		1.7	4
Rivaroxaban	ROCKET-AF (24)	3.3	2.5*
Apixaban	ARISTOTLE (32)	3	10.4

* The ROCKET-AF study reported all major bleeding episodes along with “clinically relevant” non major episodes
